# Beyond equipment distribution in Needle and Syringe Programmes: an exploratory analysis of blood-borne virus risk and other measures of client need

**DOI:** 10.1186/s12954-016-0107-0

**Published:** 2016-05-31

**Authors:** Carla Treloar, Limin Mao, Hannah Wilson

**Affiliations:** Centre for Social Research in Health, UNSW, Sydney, 2052 NSW Australia

**Keywords:** Needle and Syringe Programme, Blood-borne virus risk, Injecting drugs, Equipment reuse

## Abstract

**Background:**

Despite high levels of equipment distribution through Needle and Syringe Programmes (NSPs) in Australia, the levels of reuse of equipment among people who inject drugs remain concerning. This paper used an exploratory analysis to examine the needs of NSP client that could be addressed by NSPs to enhance service impact and blood-borne virus risk practices.

**Methods:**

People who inject drugs were recruited from six NSP sites in Sydney, Australia, to undertake a self-completed survey.

**Results:**

Using the responses of 236 NSP client participants, three factors were identified in an exploratory factor analysis: recent risky injection (Eigenvalue 3.63, 20.2 % of variance); disadvantage and disability (Eigenvalue 2.26, 12.5 % of variance); and drug use milieu (Eigenvalue 1.50, 8.4 % of variance). To understand the distribution of these factors, the standardised factor scores were dichotomised to explore those participants with ‘above average’ vulnerability on each factor. A small group of NSP clients reported a cluster of vulnerability measures. Most participants (55.5 %) reported vulnerability on none or only one factor, indicating that 45.5 % could be considered as having double (35.6 %) or triple (8.9 %) vulnerability.

**Conclusions:**

These results challenge NSPs to understand the heterogeneity among their client group and develop programmes that respond to their clients’ range of needs beyond those immediately associated with blood-borne virus (BBV) risk. This paper contributes to the growing evidence base regarding the need for BBV prevention efforts to examine strategies beyond equipment distribution.

## Background

A key response to the transmission of blood-borne viruses (BBVs) among people who inject drugs (PWID) is the provision of sterile injecting equipment [1, 2]. Providing injecting equipment, while necessary, does not adequately address all of the needs of PWID. However, there is little guidance in the literature on the extent and variety of Needle and Syringe Programme (NSP) client needs and the association of these with BBV risk practices. In the field of HIV and injecting drug use, where the evidence is strongest, combined prevention interventions are considered as the provision of sterile injecting equipment, opiate substitution treatment and access to HIV treatments [3]. These interventions, while undoubtedly important, cannot address the broader range of variables shown to impact BBV risk and cannot account for inter-relationships between these variables. This leaves NSPs potentially delivering less than optimal services if their service delivery model concentrates solely on access to sterile injecting equipment without considering broader client needs, particularly in a context such as Australia with high levels of distribution of equipment.

Australia has world-leading rates of equipment distribution for PWID [4]. However, reuse of injecting equipment remains a critical concern even among PWID attending NSPs. Approximately one in four Australian NSP clients reused their own equipment in the previous month, about 16 % shared others’ needles and syringes (receptive needle and syringe sharing) and nearly 30 % shared others’ injecting equipment (receptive equipment sharing) in the previous month [5]. Such sustained levels of unsafe injection could be partially reduced by further increasing syringe coverage as evidence suggests that approximately one in five Australian NSP clients [6] and around one-third of Australian NSP pharmacy scheme attendees [7] do not have adequate sterile injecting equipment. It is obvious that other factors, over and above the availability of sterile injecting equipment, must also contribute to the continued practices of sharing of needles, syringes and other injecting equipment among PWID [7].

How should NSPs increase their effectiveness in service delivery in order to prevent transmission of hepatitis C and other BBVs in a context of high levels of equipment distribution? The risk environment framework has generated a body of research addressing the physical, social, economic and policy factors that not only enable PWID to enact safe injecting practices [8–12] but also enhance the effectiveness of NSP service provision [13–17]. A range of factors has been found to impinge on the capacity of PWID to reduce a broad spectrum of injection-related injuries and harms. The key factors in the literature include socioeconomic disadvantage (poverty, unemployment, homelessness and dependence on social welfare dependence), health constraints due to both physical and mental illnesses [18–26] and cognitive-behavioural factors [27, 28], as well as injected-related contextual factors (e.g., injecting in public spaces) [29–31]) and peer networks [32–34].

The development and current implementation of NSP policies and service delivery models in Australia, however, has primarily drawn upon BBV prevention principles that privilege increased equipment distribution rather than addressing a range of client-prioritised needs [35–38]. In this exploratory paper, we aim to better understand how NSPs could potentially enhance their service impact by addressing a range of client-focused needs. To achieve this, rather than adopting the more conventional approach of soliciting independent predictors of unsafe injection, we used an innovative client segmentation approach to investigate factors empirically differentiating NSP clients on the basis of their potential service needs including but not limited to obtaining sterile injecting equipment.

## Methods

Convenience sampling was used across six NSP sites within a region of Sydney, Australia. The recruitment sites, which were purposefully selected to represent the range of publicly funded NSP services in this area, included two primary NSPs (stand-alone services with specialist staff) with co-located vending machine sites, two primary NSP-only sites, and two secondary NSPs (where equipment distribution is performed by staff in another health services such as community health or sexual health).

All clients who attended one of six NSP sites over the study period from October 2012 to February 2013 were eligible to participate. On the days that researchers were present, NSP staff informed clients about the study and then directed interested clients to on-site researchers who explained to clients what the study was about and provided them with an information sheet to read. At the vending machine sites, a flyer was posted on the machine and the two researchers invited potential candidates to participate once they had accessed their equipment. In addition, a small number of participants were referred to the study by their peers.

### Ethics, consent and permissions

Consenting clients completed the survey either on a touchscreen computer within the NSP or on paper and returned by mail. Participants were provided with $20 voucher to compensate them for their time. The study had ethics approval from the Human Research Ethics Committee at the University of New South Wales (HC12128) and relevant health authorities (2011/11/4.6/(3413)).

### Survey instruments and measurements

#### Risky drug injection practices

Risky injecting practices in the past month was measured by eight recoded, binary variables: (1) reuse of any needles or syringes (used by oneself or others); (2) receptive injection (injected by others rather than self-injection); (3) receptive sharing of any needles or syringes; (4) receptive sharing of any injecting equipment other than needles or syringes; (5) distributive sharing of any needles; (6) distributive sharing of any injecting equipment other than needles or syringes; (7) injection at least once per day; and (8) injection at public spaces (e.g., toilets or parks).

#### Demographic, general health and wellbeing and injection-specific indicators

Apart from age, gender, country of birth (recoded into Australian-born vs. other), sexual minority status (recoded into heterosexual vs. other), Aboriginal or Torres Strait Islander (ATSI) status (yes or no) and highest level of education attainment (recoded into up to year 10 high school only vs. other), key demographic indicators included: social welfare dependence (recoded into social welfare being the main source of current income vs. other); and history of imprisonment (yes or no).

Key indicators of general health and wellbeing included: poor self-rated health (recoded into being ‘fair’ or ‘poor’ vs. other); daily life stress (recoded into having more than two major life stressors in the past 12 months vs. other); history of any diagnoses of mental illness ever (yes or no); and existence of comorbidities (i.e., chronic physical or mental complaints) other than hepatitis C infection (yes or no).

Injection-related specific indicators included a history of injection-caused injuries not related to hepatitis C (recoded into having more than two health problems vs. other); perceived current difficulties in managing drug use (yes or no); having ‘some’, ‘most’ or ‘all’ friends being PWID (yes or no); and having spent ‘some’, ‘most’ or ‘all’ current free time with other PWID (yes or no).

### Data analysis

To identify major clustering variables that potentially differentiated participants, exploratory, latent factor analysis was used in the first step. Based on our extensive knowledge from the literature and previous findings from this project [39–41], 18 dummy coded variables (Table 1) were selected for exploratory factor analysis. In particular, these 18 indicator variables were chosen as they directly matched with major services that are or could be provided or facilitated by NSPs, namely, supply of clean needles, syringes and other injecting equipment, management of drug addiction (including treatment), peer education/support, provision of primary health care and referral to other human services. Of note, age, gender, country of birth, sexual minority, indigenous status and education were deliberately excluded from factor analysis as these six variables were considered as less modifiable demographic characteristics. The factor analysis used a principle component extraction method, followed by a Varimax rotation and a Kaiser Normalisation procedure. To be parsimonious, the threshold of keeping extracted variables in the final factors was set where the minimum factor loadings were 0.40 and minimum Eigenvalue was 1.5. Standardised factor scores (i.e., mean = 0, sd = 1), produced by the factor analysis, were used in the next step. For clustering purposes, each factor score was further dichotomised into >0 and ≤0 (as weighted sums) to indicate substantial differences between participants according to each extracted factor. In the final step, participants were clustered according to different combinations of dichotomised factor scores, reflecting the range of NSP client respondents with various degrees of injection and non-injection-related vulnerabilities. All data analysis was performed in IBM SPSS Statistics 22.

**Table 1:** Key factors that differentiated the sample

	Whole sample (*n* = 236)		Factor 1^a^ ‘recent risky injection’	Factor2^b^ ‘disadvantage and disability’	Factor 3^c^ ‘drug use milieu’
	*n*	(%)			
1. >2 major life stressors in the past 12 months^d^	96	40.7			
2. ‘Some’, ‘most’ or ‘all’ current friends being PWID^d^	140	59.3			
3. Any current, non-hepatitis C, chronic comorbidity	129	54.7		0.672	
4. Ever diagnosed with a mental illness	141	59.7		0.567	
5. Social welfare as a main source of current income	171	72.5		0.545	
6. >2 injection-caused injuries ever	94	39.8		0.498	
7. Current health being only ‘fair’ or ‘poor’	80	33.9		0.469	
8. Perceived current difficulties in managing drug use	130	55.1			0.528
9. Spending ‘some’, ‘most’ or ‘all’ current free time with other PWID	125	53.0			0.493
10. Ever imprisoned	153	64.8			0.485
In the past month					
11. Distributive sharing of any other injecting equipment^e^	66	28.0	0.722		
12. Distributive sharing of any needles	47	19.9	0.715		
13. Receptive needle or syringe sharing	47	19.9	0.640		
14. Receptive injection (i.e., injection by others)	47	19.9	0.585		
15. Drug injection at any public spaces (e.g., toilets or parks)	64	27.1	0.525		
16. Receptive sharing of any other injecting equipment^e^	91	38.6	0.499		
17. Reuse of any (prior used) needles or syringes	73	30.9	0.475		
18. Injection ≥1 per day	101	42.8	0.437		

^a^Explained 20.2 % of total variance; Eigenvalue (rotated solution) = 3.63

^b^Explained 12.5 % of total variance; Eigenvalue (rotated solution) = 2.26

^c^Explained 8.3 % of total variance; Eigenvalue (rotated solution) = 1.50

^d^Dropped from factor analysis

^e^Excluding needles or syringes

## Results

### Demographic indicators

Of the 236 participants included in the analysis, 47.9 % (*n* = 113) was recruited from Primary NSP with co-located vending machine sites, a further 30.9 % (*n* = 73) from stand-alone Primary NSP sites and the rest (*n* = 50, 21.2 %) from secondary NSP sites.

The overall participant profile has been published elsewhere [41]. Briefly, close to two-thirds of the sample were male (*n* = 153) aged 39 years (sd = 9.5) and were predominately Australian-born (*n* = 212, 89.9 %). A small proportion self-identified as being other than heterosexual (*n* = 28, 11.9 %) or being indigenous (*n* = 52, 22.0 %; noting that this is an over-representation based on general population). This group had other notable sociodemographic disadvantage. As a group, the education attainment level was low where only 55 participants (23.3 %) had completed high school beyond year 10 (i.e., more than 10-year formal schooling), over 70 % of participants (*n* = 171) reported government subsidies as the main source of their current income and close to two thirds reported a history of imprisonment (*n* = 153, 64.8 %) (Table 1).

### General health and wellbeing indicators

Apart from varied degrees of sociodemographic disadvantage, participants’ health and wellbeing were also sub-optimal. As shown in Table 1, a third (*n* = 80, 33.9 %) rated their own health being generally ‘fair’ or ‘poor’, over half of participants reporting ever being diagnosed with a mental illness (*n* = 141, 59.7 %) or having a current comorbidity (physical or mental) unrelated to hepatitis C infection (*n* = 129, 54.7 %). In the previous 12 months, of the 14 listed major life stressors in the survey, the three most common ones were (in descending order): any alcohol or drug problems (*n* = 125, 53.0 %); trouble with the police (*n* = 76, 32.2 %) and mental conditions (*n* = 69, 29.2 %). As the median of total stressors was 2 (mean = 2.59; sd = 2.09; min = 0; max = 10), the mental stress indicator used 2 as the cut-off (i.e., median-split) whereby approximately 40 % (*n* = 96) reported more than two stressors in the previous 12 months (Table 1).

### Injection- specific indicators

The average age of initiation was 19.9 years (sd = 7.0) with a majority (*n* = 218, 92.4 %) having injected in the previous month. As shown in Table 1, over half of participants (*n* = 130, 55.1 %) perceived difficulties in managing drug use by responding ‘strongly agree’ or ‘agree’ to the statement ‘I find it difficult to manage my drug use in everyday life’. Second, of the list listed injuries ever caused by drug injection, the four most commonly reported ones were (in descending order): collapsed veins (*n* = 123, 52.1 %); scarring or bruising (*n* = 120, 50.8 %); swelling of the hands or feet (*n* = 90, 38.1 %); and localised infections (e.g., abscesses or cellulitis, *n* = 87, 36.9 %). As the median of total injuries was 2 (mean = 2.20; sd = 1.82; min = 0; max = 8), the injection-caused injury indicator used 2 as the cut-off (i.e., median-split) whereby approximately 40 % (*n* = 94) reported more than two injuries ever (Table 1). Furthermore, this group had extensive social connections with other PWID, which was indicated by over half of participants having ‘some’, ‘most’ or ‘all’ friends being PWID (*n* = 140, 59.3 %) and having spent ‘some’, ‘most’ or ‘all’ free time with other PWID (*n* = 125, 53.0 %) (Table 1).

Eight indicators of risky injection practices were used in the survey. In the previous month, as shown in Table 1, the most commonly reported risky practice was injection at least once per day (*n* = 101, 42.8 %); followed by receptive sharing of any injecting equipment other than needles or syringes (*n* = 91, 38.6 %) and reuse of any needles or syringes that had been used by oneself or others prior (*n* = 73, 30.9 %). Risky injection practices that were less common but still reported by a considerable proportion of participants included distributive sharing of any injection equipment other than needles or syringes (*n* = 66, 28.0 %) and injection at public spaces (*n* = 64, 27.1 %). The three least common risky practices in the previous month were distributive sharing of needles, receptive sharing of needles or syringes and receptive injection with each practice being reported by 47 participants (19.9 %).

### Extracted cluster factors

The factor analysis produced three factors from 16 variables, accounting for 41.1 % of total variance (standard Cronbach alpha = 0.72). The first factor (F1) was labelled ‘recent risky injection’ with an Eigenvalue of 3.63 after rotation and explained 20.2 % of variance. As shown in Table 1, F1 consisted of all eight risky injection indicators and were listed in a descending order based on factor loadings. The second factor (F2) was labelled ‘disadvantage and disability’ with an Eigenvalue of 2.26 after rotation and explained 12.5 % of variance. Also listed in a descending order based on factor loadings, F2 consisted of four health indicators (current comorbidity, history of any mental illness diagnoses, multiple injection-caused injuries and poor health in general) and one sociodemographic indictor (social welfare dependence). The third factor (F3) was labelled ‘drug use milieu’ with an Eigenvalue of 1.50 after rotation and explained 8.4 % of variance. Again, listed in a descending order based on factor loadings, F3 consisted of two injection-specific indicators (perceived difficulties in drug management and having spent a considerable proportion of free time with other PWID) and one sociodemographic indicator (history of any imprisonment). Having a considerable proportion of PWID friends and experiencing multiple life stressors in the previous 12 months failed to load onto any cluster factors.

### Client segmentation

**Fig. 1:**
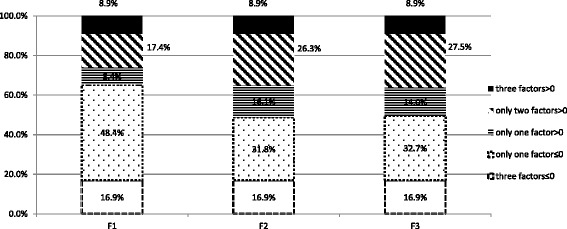
Client segmentation: indices of substantial vulnerability. *F1* recent risky injection, *F2* disadvantage and disability, *F3* drug use milieu

The three cluster factors were further dichotomised on the basis of their standardised factor scores (F1: median = −0.34; min = −1.25; max = 2.84; F2: median = −0.34; min = −1.25; max = 2.84; F3: median = 0; min = −2.73; max = 2.12, respectively). As shown in Fig. 1, for recent risky injection, 34.7 % (*n* = 82) of participants were regarded as having ‘above the average’ vulnerability. For this particular sub-group with a higher vulnerability in recent risky injection, 8.4 % (*n* = 20) had only F1 > 0 (i.e., single substantial vulnerability); 17.4 % (*n* = 41) had F1 > 0 as well as F2 > 0 or F3 > 0 (i.e., double substantial vulnerability); and 8.9 % (*n* = 21) had all three scores above 0 (i.e., triple substantial vulnerability). For social disadvantage and disability, 51.3 % (*n* = 121) of participants were regarded as having ‘above the average’ vulnerability, which could be further divided into 16.1 % (*n* = 38) with single substantial vulnerability (i.e., only F2 > 0); 26.3 % (*n* = 62) with double substantial vulnerability (i.e., F2 > 0 plus F1 > 0 or F3 > 0) and 8.9 % (*n* = 21) triple substantial vulnerability. For drug use milieu, 50.4 % of participants (*n* = 119) were regarded as having ‘above the average’ vulnerability, which can be further divided into 14.0 % (*n* = 33) with single substantial vulnerability (i.e., only F3 > 0); 27.5 % (*n* = 65) with double substantial vulnerability (i.e., F3 > 0 plus F1 > 0 or F2 > 0) and 8.9 % (*n* = 21) with triple substantial vulnerability (Fig. 1).

In other words, according to the three cluster factors, participants could be classified into the following eight, mutually exclusive groups:

- No substantial vulnerability (*n* = 40, 16.9 % in total)
- Single substantial vulnerability (*n* = 91, 38.6 % in total), which can be further divided into three groups: recent risky injection only (*n* = 20, 8.4 %), social disadvantage and disability only (*n* = 38, 16.1 %) and drug use milieu only (*n* = 33, 14.0 %)
- Double substantial vulnerability (*n* = 84, 35.6 % in total), which can be further divided into three groups: both recent risky injection and social disadvantage and disability (*n* = 19, 8.1 %); both recent risk injection and drug use milieu (*n* = 22, 9.3 %); both social disadvantage and disability and drug use milieu (*n* = 43, 18.2 %)
- Triple substantial vulnerability (*n* = 21, 8.9 %)

## Discussion

Using exploratory latent factor analysis, this paper shows that NSP clients can be essentially differentiated on three distinctive domains: risky injection, socioeconomic disadvantage and physical and/or mental comorbidities and injection-related context/milieu. This key finding suggests that in countries like Australia where the supply of sterile injecting equipment supply is already high, there is an urgent need for NSPs to deliver more client-oriented services that respond to clients’ real-life priorities and circumstances, even if some of these fall outside of the currently defined core business of NSP services.

In the 2014 WHO guidelines on HIV prevention, diagnosis, treatment and care for key priority groups, including people who inject drugs, attention was paid to the prevention and management of other co-infections and comorbidities, including mental health conditions [42]. While this is a welcome advance in understanding, other life priorities, screening and management of comorbidities such as mental health were not positioned as associated with risk of HIV transmission (in terms of practice) but as related to adherence to HIV treatment. This reflects the nascent literature in relation to understanding broader factors that can influence risk practice among PWID.

In this paper, just over one third of NSP clients (*n* = 82, 34.7 %) were classified as having substantial vulnerability in recent risky injection practices, the smallest proportion of all three domains. This is consistent with various NSP surveillance data in Australia where about one quarter to one-third PWID engage in risky injection practices during a specified period [5]. We argue that while increased distribution of sterile injection equipment is necessary, it is not sufficient to reduce risky injection practice. Indeed, these data show that measures of vulnerability relating to drug milieu and social disadvantage were larger than those relating to injecting risk, when examined individually and in combination, suggesting that clients’ concerns and needs are multiple and not necessarily prioritise BBV risk.

In attempting to understand how NSP could be provided differently and to meet client needs, we demonstrated that NSP clients could be classified into several subgroups pertaining to various degrees of vulnerability in and across each domain. This important finding challenges the conventional assumption that NSP services are best oriented towards an ‘average’ client who could be representative of the entire NSP client population [36]. Instead, our paper shows that it is important to address client heterogeneity based on broader factors that reflect social determinants of health [43] and indices of risky injection practices. Our innovative client segmentation approach based on the exploratory factor analysis revealed that while over half of NSP clients in this sample (55.5 %) could be classified as having either no substantial vulnerability across all three domains or only one substantial vulnerability in any of the three domains, the rest (i.e., 44.5 % of the sample) could be considered as having double or triple substantial vulnerability. For future NSP service modelling, policy making and resource allocation planning, our findings highlight the importance of taking into account not only clients with less demanding service needs (that is, 55 % of this sample) but also those with chronic and more complex needs, particularly those with substantial vulnerabilities on aspects of life not related only to injecting drug use.

This paper also sheds new light on the clustered rather than randomly distributed nature of key indicators. Previous findings from this project demonstrated a significant relationship between recent risky injection practices and perceived discrimination against PWID by NSP workers [41]. We argued that this relationship could be mediated by mental health, that is, the synergistic relationship between perceived stigma/discrimination and mental health is important in understanding BBV risk practice. In the current paper, we demonstrated that of those classified as having substantial vulnerability in recent risky injection, a majority (62 out of 82 participants) were also substantially vulnerable in the socioeconomic and health domain and/or in the drug use milieu domain (for example, involved in condensed PWID networks). More importantly, for clients with substantial vulnerability across all three domains (8.9 %), NSPs could provide or facilitate better client-oriented services that also support efforts to reduce BBV risk in the long-run.

There are a number of limitations to this study. Our survey was conducted in one region of Sydney with about 80 % of participants recruited from Primary NSP sites. Therefore, the findings from this convenience sample are not generalisable to other PWID, particularly those who do not regularly attend NSP services or other sites with different models of NSP delivery. The sample size may have limited the statistical power to explore other measures and detect more nuanced differences. For future research, a larger sample size and more comprehensive measures should be considered to further illuminate service needs and broader issues affecting the lives of PWID. For example, measures of daily life stressors in the past 12 months could not capture experiences of trauma during early childhood or sustained through adulthood.

## Conclusions

For more than two decades, Australia has maintained a network of publicly funded and other privately operated NSP services with a focus on dispensing sterile injecting equipment, accompanied by BBV-related health promotion. Despite this, sharing of injecting equipment has persisted at concerning levels [5]. This paper supports the growing body of literature suggesting that expanding the volume of equipment distribution alone will not be sufficient to eradicate risky injection [7, 25, 26]. This paper extends the literature by pointing out the important role that more client-tailored NSP service provision can play to effectively reduce BBV transmission by further addressing critical social disadvantage and improving adverse health and wellbeing status.

## Abbreviations

BBV, blood-borne viruses; NSP, Needle and Syringe Programmes; PWID, people who inject drugs
